# Increasing pupil size is associated with improved detection performance in the periphery

**DOI:** 10.3758/s13414-021-02388-w

**Published:** 2021-11-24

**Authors:** Lisa Valentina Eberhardt, Christoph Strauch, Tim Samuel Hartmann, Anke Huckauf

**Affiliations:** 1 General Psychology, Faculty of Engineering, Computer Science and Psychology, Ulm University, Albert-Einstein-Allee 47, 89069 Ulm, Germany; 2 Experimental Psychology, Helmholtz Institute, Utrecht University, Utrecht, the Netherlands

**Keywords:** Pupil size, Pupillometry, Peripheral vision, Detection performance

## Abstract

Visible light enters our body via the pupil. By changing its size, the pupil shapes visual input. Small apertures increase the resolution of high spatial frequencies, thus allowing discrimination of fine details. Large apertures, in contrast, provide a better signal-to-noise ratio, because more light can enter the eye. This should lead to better detection performance of peripheral stimuli. Experiment 1 shows that the effect can reliably be demonstrated even in a less controlled online setting. In Experiment 2, pupil size was measured in a laboratory using an eye tracker. The findings replicate findings showing that large pupils provide an advantage for peripheral detection of faint stimuli. Moreover, not only pupil size during information intake in the current trial *n*, but also its interaction with pupil size preceding information intake, i.e., in trial *n*-1, predicted performance. This suggests that in addition to absolute pupil size, the extent of pupillary change provides a mechanism to modulate perceptual functions. The results are discussed in terms of low-level sensory as well as higher-level arousal-driven changes in stimulus processing.

## Introduction

The light reflected by an object enters our body via an aperture in our eyes, the pupil. Interestingly, pupil size changes not only accompany changes in illuminance and within the near-triad, but also fluctuations in central arousal. As such, pupil sizes co-vary with a variety of cognitive processes, spanning such diverse phenomena as emotional activation, mental effort, or making simple decisions (see Einhäuser, 2017 and Mathot, 2018). Pupil size determines how much light enters the retina. Thus, changes in pupil size result in changes in optics. This raises the question of which perceptual consequences arise from the covariation of pupil size with numerous causes. The present paper presents two experiments addressing this question, using brightness-induced pupil size changes.

Pupils adjust to provide the optimal visual image quality, when co-varying with brightness as well as with changes within the near-triad (e.g., Campbell, 1957; Campbell & Gregory, 1960; Charman & Whitefoot, 1977; Crawford, 1936; Feil et al., 2017; Woodhouse, 1975). Pupillary changes also co-occur with changes in arousal. More specifically, pupil size is demonstrated to reflect changes in activation in the locus-coeruleus (LC) and the associated noradrenergic system (Joshi et al., 2016; Murphy et al., 2014). Further, microstimulation of the superior colliculus (SC) elicits changes in saccade and pupil responses in monkeys, whereas changes in luminance only affect pupil size changes. This suggests that the SC might be coordinating non-luminance-linked input to the LC (Wang & Munoz, 2021). Tonic and phasic activity of the LC can be dissociated, with tonic activation being linked to tonic firing in the LC, in common experimental paradigms thus usually reflecting baseline activation, and phasic activation, linked to phasic firing in the LC, usually reflecting task-evoked pupillary responses. Due to its link to various cognitive processes, it has been assumed that LC activation, which becomes visible in pupil size, mediates cognition (Sara & Bouret, 2012).

Changes in pupil size are often accompanied with differential (visual) task performance. Respective changes have frequently been attributed to differences in attentional processing (e.g., Brocher et al., 2018; Klatt et al., 2021; Unsworth & Robison, 2016; Van Kempen et al., 2019). However, a recent hypothesis suggests that the function of arousal-linked pupillary changes is to tune vision at the point of initial sensory intake to improve perception (Ebitz & Moore, 2019; Mathôt, 2020; Mathôt & Ivanov, 2019). This would imply that visual task performance is in some cases not only affected by central activation, but also by mere sensory information.

Based on optics, the aperture of the eye, that is, the pupil’s size, is expected to affect the visual image. Small apertures focus the incoming light rays more, reduce retinal light scatter, and eliminate optical aberrations of the peripheral parts of the eyes’ cornea. In sum, small apertures provide a better resolution of high spatial frequencies, and, thus, allow discrimination of fine details. Large apertures, in turn, allow more light to enter the eye, thus providing a better signal-to-noise ratio, though at the cost of high resolution. Therefore, with large pupils only low spatial frequencies can be resolved. However, visual sensitivity, that is, detecting whether there was a signal at all, is improved with large pupils (e.g., Artal, 2014; Ebitz & Moore, 2019; Mathôt & Ivanov, 2019).

These assumptions based on the optics of the eye are widely supported by optometrical studies on the impact of pupil size on visual acuity. For example, Campbell and Gubish (1966) examined the resolution of the light reflection from the fundus in the eye. They demonstrated that the most detailed profile of the light reflection results from small pupils, concluding that small pupils increase visual acuity. Further, Campbell and Gregory (1960) could demonstrate that pupil size at a given brightness enables an optimal optical resolution for high contrast stimuli. Participants’ eyes were enlarged with homatropine; they then looked through artificial apertures of different diameters while they had to adjust the ambient brightness until they were able to discriminate grid patterns. The authors compared these results to those obtained with natural pupil sizes at a given ambient brightness. It turned out that, for a given brightness, those artificial pupil sizes leading to an optimal optical resolution resembled natural pupil sizes. Campbell and Gregory (1960) therefore considered the pupil light response to have a crucial function in enabling optimal optical resolution for a broad range of brightness. Woodhouse (1975) replicated and extended these findings, showing that the same effect holds not only for high-contrast stimuli, but also for a broad range of contrast levels.

Besides such optometric studies, there is renewed interest in the interplay between pupil size and perception from a psychological point of view in some recent works. These works suggest that not only brightness-related but also arousal-linked pupil size changes adjust vision to subserve task-relevant goals. Ebitz and Moore (2019) review evidence on the impact of cognition on the pupillary light response, as well as on the impact of cognition on pupillary responses for constant luminance conditions. They suggest that pupillary changes can not only be regarded as a gauge for cognitive processes, but could also act as a filter for basic visual information intake to contribute to an optimization of visual perception for particular goals. In a recent review, Mathôt (2020) went even further, referring to arousal-linked pupil size changes as a form of sensory tuning for the current and immediately following situation. He emphasizes the role of pupil constrictions, which is of special importance for foveal vision, since it enhances visual acuity. This is supported by recent empirical evidence from Mathôt and Ivanov (2019). In two experiments a foveal discrimination task as well as a peripheral detection task in which faint stimuli were presented at 7.7° eccentricity were conducted. Pupil size was manipulated by varying the brightness of the task-irrelevant further visual periphery (above 25°). For foveal vision they found improved visual discrimination performance with a bright background, but only in a third experiment in which stimuli of near-threshold size were used. This suggests that small pupils can enhance visual discrimination at the focus, although respective effect sizes seem to be small. Detection performance in the visual periphery was robustly better with a dark compared to a bright background in both experiments. Interestingly, similar to task performance, pupil size was predicted not only by brightness, but also by task: pupil size was larger during the detection task compared to the discrimination task although both tasks were equally difficult. Thus, detection is improved for a dark background and pupils are larger during a detection task. This strongly suggests that detection performance is improved with large pupils. However, the authors did not ultimately test whether pupil size directly could predict detection performance.

Woodhouse and Campbell (1975) investigated various proposals for the function of the pupillary light reflex and demonstrated that pupil dilation improved visual sensitivity. Further, their results show that detection thresholds improve faster with dark adaptation when the pupil during a preceding light-adaptation period was naturally constricting compared to being artificially fixed at a dilated state. Woodhouse and Campbell (1975) concluded that the naturally adapting pupil size when constricting can attenuate the bleaching of retinal cells by reducing retinal illumination during light adaptation to support visual sensitivity in response to subsequently occurring darkness. Especially the rods in the retina should benefit from dynamically adapting pupil size, since they take a long time for dark adaptation. Thus, reducing effects of light adaptation by constricting the pupil restores visual sensitivity for a return to darkness. Since the rods are distributed primarily in the peripheral parts of the retina, perceptual effects of dynamically adapting pupils should be observable especially in peripheral vision.

Taken together, it is still unclear whether alterations in task performance are to be attributed to fluctuations in central activation (as indicated by pupil size; e.g., Van Kempen et al., 2019; Unsworth & Robison, 2016) or to changes in sensory information since pupil size changes alter retinal illumination, resulting in an altered visual image. The above reviewed evidence shows that small compared to large pupils improve visual acuity, which is of importance for foveal vision. Further, there is some evidence that large pupils improve visual sensitivity, measured, for example, by detection performance. Since this seems to be especially related to dark adaptation by the rods in the retina (Woodhouse & Campbell, 1975), this should be observable especially in peripheral vision (i.e., Mathôt & Ivanov, 2019; Weiler, 1910). However, evidence for effects of pupil size on peripheral vision is still weak. In the present study, we investigate the impact of pupil size, manipulated by brightness of a task-irrelevant background, on peripheral detection performance.

Interestingly, also for arousal-linked pupillary changes, the role of pupil dynamics, including tonic and phasic components, becomes more and more evident. Performance on a set of tasks cannot only be predicted from the pupil diameter observed during a task, but also from pupil diameter *preceding* a task, usually during baseline. Schriver et al. (2020), for instance, reports an association of baseline pupil size and response times during subsequent trials in animal experiments. However, Hong et al. (2014)) instead find no such link in humans using an auditory oddball task, similar to Beatty (1982). Van Kempen et al. (2019) find pupil size during a perceptual decision-making task, but also pupil size during baseline, to be predictive for task performance. Here, participants had to press a button as soon as previously randomly moving dots in two peripheral patches started to coherently move into one direction. Dots were presented at an eccentricity of 10° of visual angle to the side and 4° down relative to a central fixation dot. Pupil size was twofold associated with reaction time: First, larger pupil sizes during baseline were associated with slower reaction times during the subsequent trial. Second, the larger the increase in pupil size during the trial, the faster the reaction time. These effects were accompanied by changes in brain activity as measured with an EEG: Higher baseline arousal was associated with higher variability in responses to targets; trials with a larger increase in pupil size instead were associated with less variability in the electrocortical response. It is important to note, however, that a differentially large pupil size during a visual task, as in Van Kempen et al. (2019), likely also may have caused a systematically different visual sensation. Hence, the apparently conflicting findings (Hong et al., 2014 and Van Kempen et al., 2019) could thus be the result of the modality (visual vs. auditory) used for the task: Whereas pupil size changes affect the processing of light and thus influence the visual performance (as in Van Kempen et al., 2019), they are not involved in the processing of sound waves and thus do not alter measures of auditory performance (as in Hong et al., 2014, and Beatty, 1982).

Taken together, beyond the pupil size during information intake, pupil size preceding information intake might also be informative about task performance. This could either be due to a sensational effect of differentially large pupil sizes during baseline/the foregoing trial, or due to differential arousal states associated with such differential large pupil sizes. Based on findings by Woodhouse and Campbell (1975), as well as by Van Kempen et al. (2019), it can thus be hypothesized that smaller pupil sizes during a foregoing trial would be associated with improved performance on the next trial.

In the present study, we examined two questions: First, the reliability of reported perceptual changes with altered pupil size, and, second, effects of current and preceding pupil sizes on detection of faint peripheral stimuli. Therefore, we manipulated the brightness of a task-irrelevant background. Experiment 1 was conducted as an online study. This should put the hypothesis that peripheral detection performance is increased with large pupil size to a difficult test, because neither the ambient lighting condition nor the screen brightness could be fully controlled (e.g., due to different devices with varying screen sizes). Based on previous evidence, we assumed additionally that the probability of detecting the stimulus increases with larger pupil size. Thus, in Experiment 2, an eye-tracker study, we replicated Experiment 1 and additionally measured pupil size. To determine the effects of pupil dynamics, we modeled the data to include not only pupil size during information intake but also the pupil size during the previous trial into the analysis.

## Experiment 1: Online experiment

### Methods

#### Participants

This research was conducted according to guidelines of the German Psychological Society (DGPs), the German Research Foundation (DFG), and in line with the Declaration of Helsinki. Before the experiment was performed, written informed consent was obtained. In total 47 participants started the experiment. They were recruited online from the community of Ulm University and via personal request. If they wished, participants could receive partial course credit. Complete datasets of *n* = 31 participants (*M*_*age*_ = 23.0 years, *SE* = 0.49) were gathered and included in the statistical analysis.

#### Apparatus

The experiment was conducted as an online study. For the implementation PsychoPy and PsychoJS (Version 2020.1.2) were used and the experiment was hosted on Pavlovia. Participants received the link to the experiment as part of a personal contact or via the experiment-management webpage of Ulm University.

Implementing the stimulus properties, the experiment was piloted on a Macbook Air 2015 13-in. device with a 1,440 × 900 px screen and 60 Hz. To achieve comparable testing conditions for all participants, detailed instructions were provided during the course of the experiment: Participants were instructed to position themselves 50 cm from their screen throughout the experiment and to face it full on. The monitor brightness was to be at its maximum, the shutters of the room closed, and the lights of the room turned off. The experiment was only to be conducted during the day. Participants with poor eyesight were to wear their prescribed visual aid.

#### Stimuli and design

**Fig. 1:**
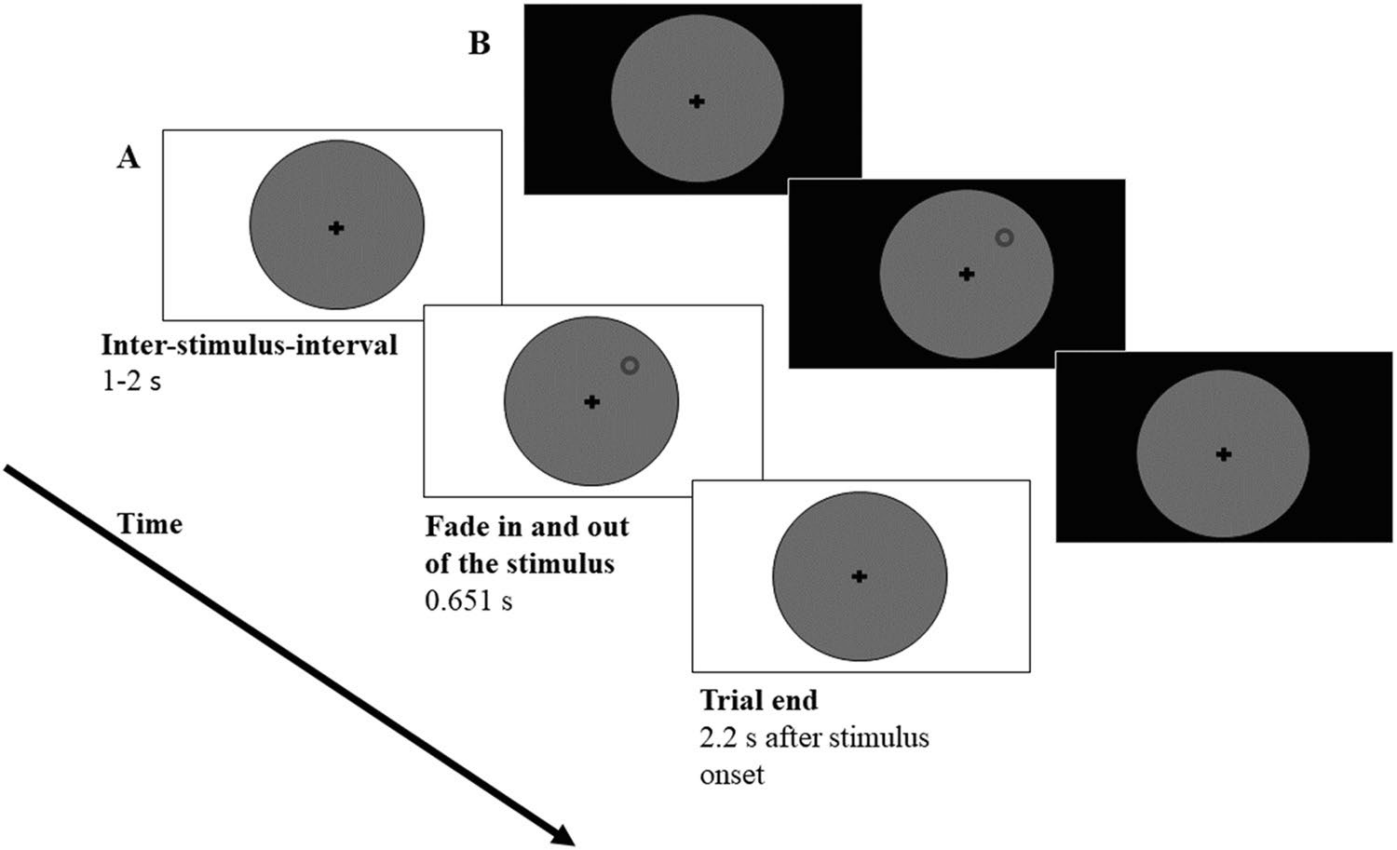
(**A**) Sequence of a trial with bright background brightness and stimulus position at 45°. (**B**) Sequence of a trial with dark background brightness and stimulus position at 45°. For each trial background brightness, stimulus position, and stimulus intensity were chosen in random order. After the start of a trial there was an inter-stimulus interval of 1–2 s. Subsequently the stimulus was faded in and out linearly for 0.651 s. The trial ended 2.2 s after stimulus onset

Screen size and viewing distance were estimated for each participant using the virtual chinrest procedure described by Li et al. (2020) to adjust stimulus properties for each participant individually in terms of visual angle. In this procedure, the physical size and resolution of the individual device is measured by asking participants to adjust a rectangle to the size of a standard credit card. In addition, participants’ blind spot, which is known to be located at an eccentricity of 15°, is measured. Given these parameters, participants’ viewing distance was calculated and stimuli were adjusted accordingly. Stimuli were presented on a local background of a gray disc (51.5 lx on the reference screen) in the center of the screen with a diameter of 26.65°, visible throughout the whole experiment (see Fig. 1). In the center of the disc, a black fixation cross (0.7 lx on the reference screen) with a size of 0.51° was presented permanently. The to-be-detected stimuli were rings with a diameter of 1.02° and a line width of 0.08°. The stimuli were presented on an imaginary circle of 7.70° at one of eight randomly chosen positions (0°, 45°, 90°, 135°, 180°, 225°, 270°, and 315°). Brightness of the task-irrelevant background was varied randomly in each trial by presenting either bright (white, 300 lx on the reference screen) or dark (black, 0.7 lx on the reference screen) background (see Fig. 1). To-be-detected stimuli were presented with four stimulus intensities of increasing intensity (referred to by 1–4 in the following). Thereby, we ensured that for each participant, despite different presentation conditions due to the online setting, in some trials uncertainty for detection occurred. Manipulating background brightness (bright, dark), stimulus intensity (absent, 1–4) and stimulus position (0°, 45°, 90°, 135°, 180°, 225°, 270°, and 315°) with each of these conditions repeated twice resulted in a total of 2 (background brightness) ⨯ 5 (stimulus intensity) ⨯ 8 (position) ⨯ 2 (repetitions) = 160 trials.

#### Procedure

##### Overview

The link to the experiment was shared in the experiment-management system of Ulm University or via direct mail contact.

The experiment started with information about the study and the written informed consent, and continued with the estimation of the viewing distance by using the virtual chinrest procedure from Li et al. (2020). First, to record screen resolution, participants adjusted the size of a rectangle on the screen to the physical size of a credit card. Second, the blind spot was measured five times to receive a reliable estimate of the participant’s viewing distance by using the mean of these five measures. If the variance of the five values was larger than 60 px, the process had to be repeated. Using this information, all stimuli for the rest of the experiment were adjusted in terms of visual angle.

For the detection task, ten practice trials were conducted in advance. The test phase comprised 160 trials. After every 40 trials, pauses were offered that had to be terminated by the participant in order to continue the experiment.

##### Detection task

Figure 1 shows the sequence of one trial. The fixation cross and the local background were presented throughout the whole experiment. In the beginning of each trial, the background brightness level was randomly chosen. Thus, background brightness either switched or remained as in the previous trial. The inter-stimulus interval (ISI) between 1–2 s was drawn randomly from an equal distribution. Afterwards, the stimulus, a gray circle of varying intensity, was faded in and out linearly for a total of 0.651 s. In more detail, the stimulus was faded in for 0.279 s, was fully visible for 0.093 s, and was faded out for 0.297 s. A trial ended 2.2 s after stimulus onset and independently of the participant’s response. Participants’ task was to fixate the fixation cross throughout the experiment and press the space bar whenever they detected a stimulus. Participants were instructed to respond as accurately as possible. Key presses in the 2.2 s after stimulus presentation were counted as correct detection. For the ten practice trials the stimulus intensity was held constant at the highest possible level.

### Results

One hundred and twenty-eight trials (excluding the stimulus-absent condition) of *n =* 31 participants led to a total of *n =* 3,968 trials to be included in the analysis. Means and standard errors for correct target detection in each condition are presented in Table 1.

**Table 1:** Means and standard errors of correct target detection as a function of previous background brightness, background brightness, and stimulus intensity

Brightness *n*-1	Dark	bright	bright	Dark
Brightness *n*	dark		Bright	
Stimulus Intensity	*M (SE)*	*M (SE)*	*M (SE)*	*M (SE)*
1	2.8% (1.1)	7.1% (2.5)	4.0% (1.2)	6.7% (1.8)
2	27.3% (5.0)	23.4% (3.8)	21.3% (4.8)	17.0% (3.7)
3	46.5% (6.2)	49.7% (5.3)	46.0% (6.1)	45.8% (5.6)
4	60.8% (5.9)	60.3% (5.8)	55.6% (5.8)	54.3% (5.8)

Detection rates increase with increasing stimulus intensity descriptively. Dark background brightness goes hand in hand with a descriptively higher detection rate in comparison to bright background brightness

To investigate the effect of stimulus intensity, background brightness *during* (i.e., in a trial *n*) and background brightness *preceding* information intake (i.e., in trial *n*-1) on peripheral detection performance on a trial basis, we conducted a logistic mixed-model analysis, which was nested within participants. The analysis was run using RStudio (Version 1.4.1717) and the package lmerTest (Kuznetsova et al., 2017). The formula for the fitted model is detection ~ intensity + brightness * preceding brightness + (1 | participant), using random intercepts for participants. The final model was chosen based on theoretical considerations and parsimony.

**Fig. 2:**
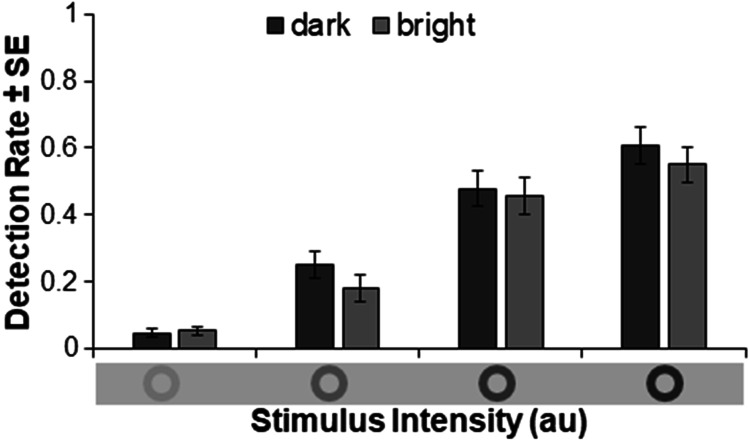
Mean detection rates and standard errors as a function of stimulus intensity and background brightness in Experiment 1

The results of the analysis presented in Table 2. Detection performance was predicted significantly by stimulus intensity (*Z* = 26.485, *p* < .001): With increased stimulus intensity the probability of detecting the stimulus is increased. Further, detection performance was predicted significantly by background brightness (*Z* = -3.212, *p* = .001). The probability of detecting the stimulus is reduced with a bright compared to a dark background by 32.1% (odds ratio (*OR*) = 1.321). Background brightness in the previous trial, as well as its interaction with background brightness (in trial *n*), did not significantly predict detection performance. In Fig. 2 detection performance is plotted as a function of stimulus intensity and background brightness.

**Table 2:** Effects of the logistic mixed model in Experiment 1

Effect	*Estimate*	*SE*	*Z*	*p*	*Odds ratio*
Intercept	-3.129	0.278	-11.241	< .001	0.044
Stimulus intensity	1.270	0.048	26.485	< .001	3.561
Background brightness (*n*)	0.279	0.087	3.212	.001	1.321
Preceding brightness (*n*-1)	0.012	0.087	0.140	.888	1.012
Brightness (*n*) * Brightness (*n*-1)	-0.150	0.174	-0.859	.390	0.905

The number of false alarms (absolute number of key presses while no stimulus was presented) was overall low: For the dark background *M*_dark_ = 3.68 (*SE* = 2.42) and for the bright background *M*_bright_ = 3.65 (*SE* = 2.16) false alarms were recorded.

### Interim discussion

The results of Experiment 1 show increased peripheral detection performance with increased stimulus intensity. Thus, the different stimulus intensities were of different degrees of difficulty for the detection of the stimuli. Moreover, as expected, stimuli were better detected when the background brightness of the participant’s screen was dark compared to bright. This replicates the findings of Mathôt and Ivanov (2019) and shows in addition that the effect reliably occurs, even in a suboptimally controllable setting such as an online study. That peripheral detection performance is better for a dark compared to a bright background is in line with the assumption that visual detection in the periphery is enhanced when pupils are larger. However, due to the online nature of Experiment 1, we can only say with certainty that background brightness is related to improved detection performance in the periphery.

## Experiment 2: Lab experiment

To investigate whether pupil size is indeed linked to the improved peripheral detection performance, we replicated the experiment in a laboratory setting, recording pupil size in addition.

### Methods

The experiment in the laboratory was a replication of the online Experiment 1 while additionally measuring the pupil size and adding a fixation control. Thus, only changes in the methods relative to Experiment 1 are described.

#### Participants

Six students from Ulm University participated in the experiment (*M*_age_ = 22.5 years, *SE* = 1.67). All participants provided written informed consent prior to participation.

#### Apparatus

A SMI HiSpeed 1250 eye-tracker (SensoMotoricInstruments GmbH) was used for obtaining pupil sizes and ensuring constant gaze position. Stimuli were presented on a BenQ 28-in. Screen (100 Hz, 1,920 × 1,080 px). Participants’ viewing distance was fixed at 50 cm using a chinrest. For 1 s before the stimulus was presented, gaze position had to stay constantly within a radius of 1.275° around screen center. In case the gaze left this area, the ISI was prolonged until fixation was stable for 1 s.

#### Data preparation

Pupil size data were measured throughout the experiment. Blinks and artifacts in the pupil data were filtered out and missing data were interpolated as described in Georgi et al. (2014). Pupil data were downsampled to 50 Hz for all further analyses. For data analysis, pupil size during a time interval starting 1 s prior to stimulus presentation (i.e., constant fixation) and lasting until the end of the stimulus presentation was averaged. Mean pupil sizes in this time interval of 1.65 s were z-standardized (mean pupil size in a trial subtracted by mean pupil size of all trials and divided by standard deviation) based on the mean value of all *n* = 768 trials (128 target-present trials × 6 participants).

### Results

**Fig. 3:**
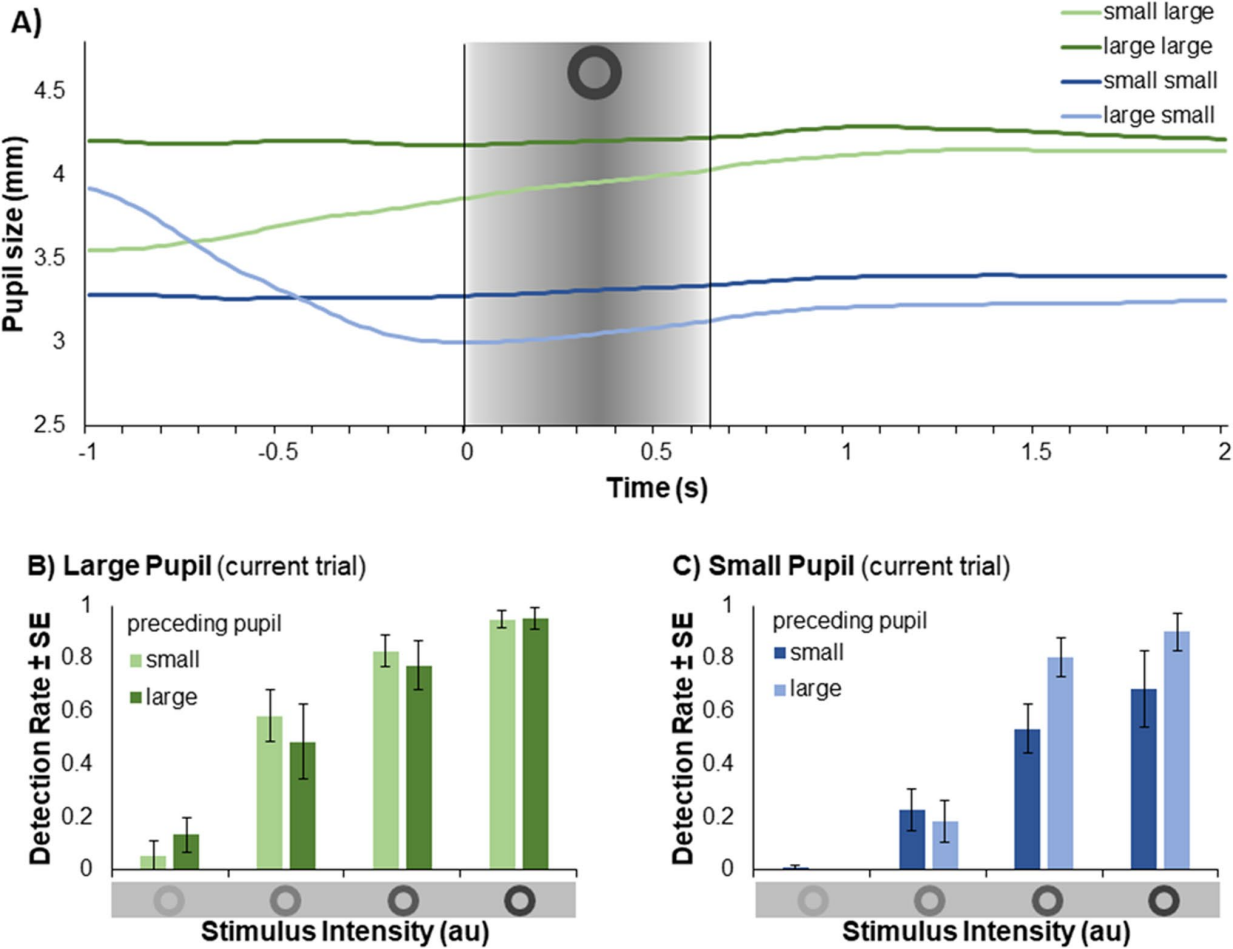
(**A**) Pupil size changes over time averaged across trials as a function of pupil size in the current trial *n* and the previous trial *n*-1, categorized by an average split. The gray area highlights the interval of 650 ms during which the stimulus was faded in and out. (**B**) Detection performance in trials with large pupil size as a function of stimulus intensity and pupil size in the previous trial. (**C**) Mean detection performance and in trials with small pupil size as a function of stimulus intensity and pupil size in the previous trial

The absolute number of false alarms (calculated as in Experiment 1) was also low in Experiment 2. For the dark background *M*_dark_ = 1.00 (*SE* = 0.45) and for the bright background *M*_bright_ = 2.33 (*SE* = 0.61) false alarms were recorded. Pupil size for the dark background was with *M*_dark_ = 4.087 mm (*SE* = 0.150) significantly larger compared to for the bright background with *M*_bright_ = 3.279 mm (*SE* = 0.113), *T*(5) = 12.123, *p* < .001. A summary of the data of Experiment 2 are plotted in Fig. 3. For the means of descriptive data, trials were categorized as a function of current pupil size in trial *n* and previous pupil size in trial *n*-1 based on an average split. Pupil size changes averaged across participants are depicted in Fig. 3A. As in Experiment 1, stimulus detection was higher with increased stimulus intensity, as well as with large compared to small pupils (see Figs. 3 B and C).

For inferential analysis, we used mean pupil size as a predictor variable in Experiment 2 instead of using background brightness and previous background brightness, because pupil size should be the proximal measure of the brightness manipulation. To investigate the effect of stimulus intensity, pupil size *during* (i.e., mean pupil size in a trial *n*) and pupil size *preceding* information intake (i.e., mean pupil size in trial *n*-1) on peripheral detection performance on a trial basis, we conducted a logistic mixed-model analysis, which was nested within participants. 768 trials were included into the analysis. The analysis was run using RStudio (Version 1.2.1335) and the package lmerTest (Kuznetsova, Brockhoff, Christensen, 2017). The formula for the fitted model is detection ~ intensity + pupil * preceding pupil + (1 | participant), using pupil size as z-standardized measures and random intercepts for participants. The final model was chosen based on theoretical considerations and parsimony.

**Fig. 4:**
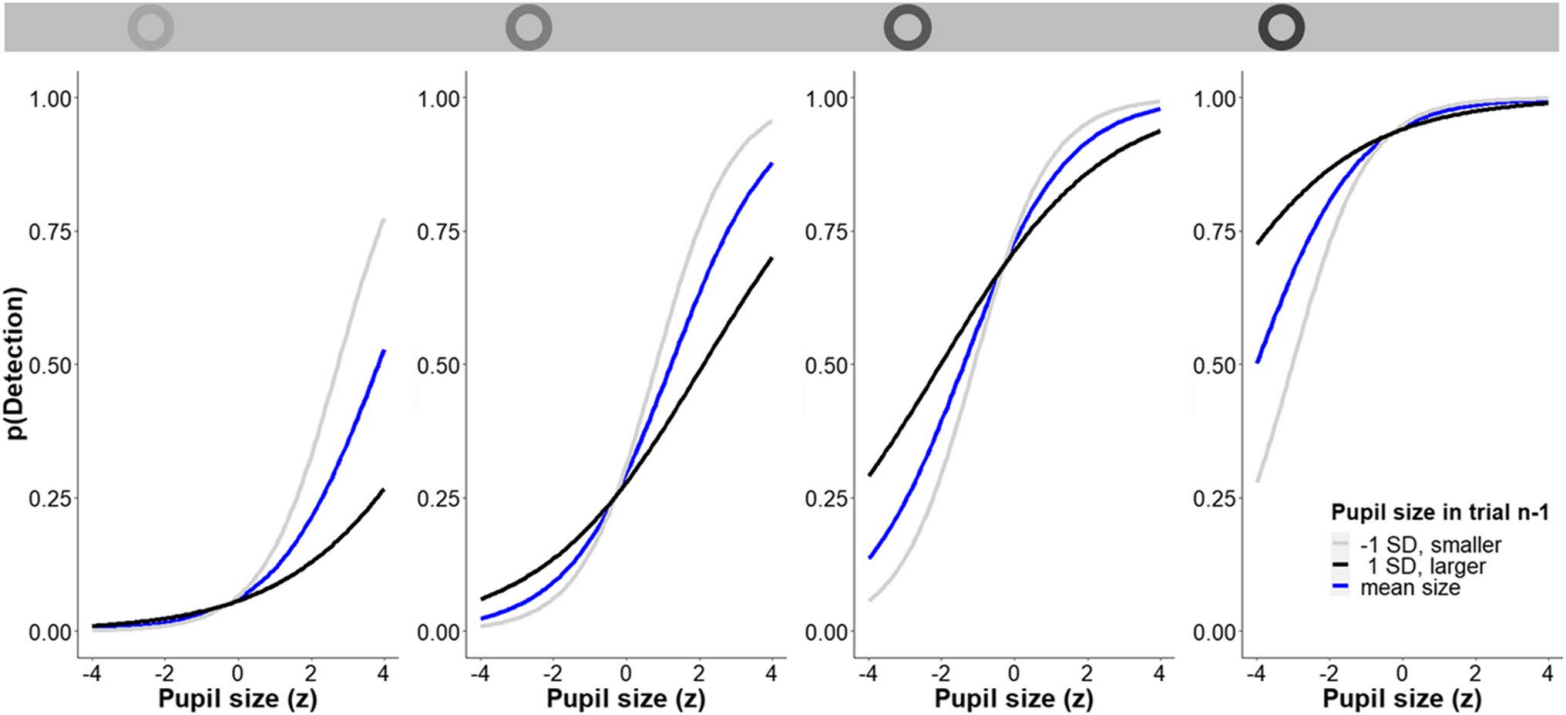
Fitted logistic mixed regression models plotted separately for the four stimulus intensities. The blue lines depict the effect of pupil size during information intake on the probability of detecting the stimulus (given a mean pupil size preceding information intake). The light gray and black lines plot the direction of the interaction between pupil size during and pupil size preceding information intake for preceding pupil sizes of 1 *SD* smaller (light gray) or larger (dark gray) than the mean pupil size

The results of the analysis presented in Table 3 show that stimulus intensity significantly predicts detection performance: With increased stimulus intensity the probability of detecting the stimulus is increased. Further, detection performance was predicted significantly by pupil size (*Z* = 4.979, *p* < .001), showing that, while holding all other predictors constant, the probability of detecting the stimulus was increased by 102.6% (*OR* = 2.026) when pupil size was increased by 1 *SD*. This effect is depicted in Fig. 4 by the blue line. The effect of pupil size in trial *n*-1 was not significant (*Z* = -0.596, *p* = .551). Detection performance was predicted significantly by the interaction of pupil size in trial *n* and pupil size in trial *n*-1 (*Z* = -3.143, *p* = .002, *OR* = 0.770). The interaction is depicted by the light gray and black lines in Fig. 4 as examples: The probability of detecting the stimulus with larger pupils is higher, the smaller pupil size was in trial *n*-1 (here detection for: 1 *SD* smaller, gray; 1 *SD* larger, black). Thus, small pupils in the previous trial enhanced the positive effect of large pupil sizes on peripheral detection performance, as indicated by steeper curves.

**Table 3:** Effects of the logistic mixed model in Experiment 2

Effect	*Estimate*	*SE*	*Z*	*p*	*Odds ratio*
Intercept	-2.729	0.397	-6.881	<.001	0.065
Stimulus intensity	1.862	0.128	14.597	<.001	6.437
Pupil (*n*)	0.706	0.142	4.979	<.001	2.026
Preceding pupil (*n*-1)	-0.077	0.128	-0.596	0.551	0.926
Pupil (*n*)*Pupil (*n*-1)	-0.261	0.83	-3.143	0.002	0.770

## General discussion

The experiments here presented investigated effects of pupil size changes on visual perception. Here, we examined the effects of pupil dilation on detection performance in the visual periphery, a phenomenon that has only been investigated in a single study so far (Mathôt & Ivanov, 2019). We conceptually replicated findings for the detection of faint stimuli in the periphery both in an online (Experiment 1) and in a laboratory (Experiment 2) experiment. The online replication in Experiment 1 indicates a stable and reliable effect. Extending these findings, we showed that detection performance is not only determined by pupil size *during* information intake, but also by pupil size *preceding* information intake.

Regarding our first contribution, detecting peripheral faint stimuli on a gray background depends on the brightness of a task-irrelevant background in farther periphery: With low background brightness, peripheral detection performance was better than with high background brightness. This effect of background brightness was observed in Experiment 1, an online study, with a large sample of 31 participants. In a controlled laboratory setting with six participants, we replicated Experiment 1 and found pupil size manipulated by background brightness to predict peripheral detection performance. Hence, one might plausibly assume that also in Experiment 1, effects were related to pupil size. While stimulus intensity, as expected, affected the difficulty of the task, no interaction between stimulus intensity and the effects of pupil size on detection performance was found. As the findings in the lab study (Experiment 2) indicate, better detection performance was associated with larger pupils during information intake. It has to be noted that the data presented here are based on a relatively small sample for Experiment 2 (but not Experiment 1). Given the consistency of findings of the experiments reported here and the previous experiment by Mathôt and Ivanov (2019), the internal replication here and the within-subjects design, we doubt that a larger sample size would alter any results. The findings are in line with earlier reported better peripheral detection performances for dilated pupils (Mathôt & Ivanov, 2019) and thus support the proposed effects of pupil size on visual information intake (Ebitz & Moore, 2019; Mathôt, 2020; Mathôt & Ivanov, 2019).

Extending this previous evidence, we found detection performance to not only be altered by the pupil size during information intake (i.e., during the trial), but also by the interaction with the pupil size preceding information intake (i.e., during the previous trial). More specifically, small pupils in the previous trial enhanced the positive effect of large pupils during information intake on detection performance. That is, detection performance was best when the pupil was large and relative to the previous trial characterized by a strong increase in pupil size (this is reflected in Figs. 3 and 4 by the steepest curves, light gray). Hence, the more pupils dilate from a baseline level, the better the detection performance, even if the previous trial is considered as baseline. It is especially remarkable that the effect of pupillary dilation (i.e., the interaction of small pupils in the previous trial and large pupils in the current trial) results in higher detection performance compared to constantly large pupils, even though the absolute pupil size *remains smaller* in trials in which pupil size has changed. Relatedly, for small pupils the significant interaction of previous pupil size and pupil size in the current trial suggests that detection performance also benefits from a change in pupil size in the case of pupillary constriction. Again, although absolute pupil size becomes smaller compared to trials in which pupils remain constantly small, *detection performance is higher* in trials in which pupil size has changed.

Taken together, the present data suggest first that absolute pupil size plays an important role for peripheral detection of faint stimuli with large pupils improving peripheral detection performance. Second, in addition to absolute pupil size – though to a smaller degree – the extent of pupillary change seems to affect peripheral detection performance: Whenever pupil size changed within the few seconds prior to stimulus presentation, detection performance was increased. The direction of pupillary change did not seem to play a role for this enhancement effect. Thus, one might speculate whether pupillary change constitutes a mechanism of reorientation for the visual system. This might work, for example, by counteracting processes of retinal adaptation by the changing retinal illumination.

To the best of our knowledge, no factors other than pupil size seem to drive the effects we report here. For example, one might wonder whether the high number of target-present trials induced a response bias that could indirectly drive effects. However, the number of false alarms was consistently very low with less than four or less than three false alarms on average in Experiment 1 and Experiment 2, respectively, with no indication for a difference between background brightness conditions.

Further, we could not find any evidence for attentional lapses to affect detection performance. Detection performance in the previous trial can be regarded as an indicator of attention to the task. An alternative model in which we considered detection performance in the previous trial as a predictor for detection performance in the current trial did not show a relation, thus arguing against strong inattention to drive the effects (for analysis details, see Online Supplementary material https://osf.io/ujbts/?view_only=b5dac82edd884c6bb861a74a6f8fdcec). Alternatively, one might argue that improved detection performance for trials in which pupil size changes (i.e., the interaction effect that we observed) are due to the fact that pupillary changes are coupled to changes of background brightness, and that these changes elicit attentional reorientation to the task. If this were the case, one would expect pre-trial effects also for brightness as a predictor. In fact, the interaction of previous background brightness and current background brightness should then predict detection performance even better than the interaction that we observed for pupil size. However, only the interaction of pupil size preceding and during information intake predicted detection performance (Experiment 2), brightness did not (neither in Experiment 1 nor Experiment 2; see Online Supplementary Material https://osf.io/ujbts/?view_only=b5dac82edd884c6bb861a74a6f8fdcec). This strongly suggests that – although brightness and pupil size are strongly coupled – pupil size is more than just a proximal measure for brightness.

We see two possible interpretations for the effects we observed considering sensory changes due to altered information intake on the one hand and arousal changes on the other hand. In terms of altered information intake, effects of pupil size on perception should occur in a very early low-level stage within the cascade of hierarchical visual feedforward processing: An enlarged aperture, that is, a dilated pupil, leads to an increased amount of light entering the eye. Thus, a larger change in pupil size could be associated with stronger physiological alterations on the very low-level retinal level, resulting in a stronger stimulation of retinal receptor cells. This should be beneficial for visual sensitivity, especially for rods densely distributed in the visual periphery (Woodhouse & Campbell, 1975). However, changes in arousal could also cause physiological alternations in early visual processing, for example, with larger dilations eliciting detection-beneficial activation in early neural connections.

Regarding arousal, larger pupil sizes could be indexing higher-level activation with higher arousal reflecting an attentional mechanism that is beneficial for detection. Vice versa, task performance might be hampered by a suboptimal arousal level during baseline. This would be in line with findings on visual task performance and arousal, as measured by pupil size interpreted as a result of fluctuations in activation in animals (Schriver et al., 2020) and humans (Van Kempen et al., 2019). However, if arousal, indicated by pupil size, affects higher-level stimulus processing, performance in other modalities should also be improved, which is not reported for the auditory domain (Beatty, 1982; Hong et al., 2014). Effects of (baseline) pupil size on visual task performance could thus be partially misinterpreted as effects of arousal, but in fact result from alternations in pupil size. Hence, future studies should explore whether pupil size at baseline can predict task performance in other modalities (such as tactile, auditory domains, etc.). While out of scope for the current paper, it would be most interesting to run a follow-up study that specifically addresses the potential link between pupil size, detection performance, and reaction time to see whether pre-trial pupil size affects response behavior (merely) via a perceptual (as indicated by our data), an attentional (as suggested by Van Kempen et al., 2019, and Unsworth & Robison, 2016), or both paths. Whether the effects reported here in fact can be traced back to low- or higher-level processing is still unclear. Further investigations of pupillometry in combination with arousal in central (e.g., EEG, see Hong et al., 2014; Murphy et al., 2011) as well as in peripheral measures (skin conductance, e.g., Ehlers et al., 2016) may help to disentangle the underlying mechanisms.

Future studies that investigate the effect of pupil size on visual task performance should also take into account other methods to manipulate pupil size in order to eliminate potential interference by visual manipulation. However, while it would be intriguing to see whether pupil size changes co-varying with emotional activation or mental effort predict detection performance in a similar way to that found here, the extent of such pupil size variation might be too small to see an effect on visual detection performance. Stimulation of the vagus nerve might provide an alternative to manipulate pupil size; however, evidence is conflicting here with many null results also reported (Mridha et al., 2021, reports such effects in mice; but see also Burger et al., 2020; Keute et al., 2019; Warren et al., 2019, for no such effects in humans). Further, since small finger movements like pressing a button already produce reliable and substantial pupil size changes (Richer & Beatty, 1985; Strauch et al., 2020), more intense movements like continuously tapping with a hand or a foot might induce sufficiently large pupil sizes.

Taken together, the current results demonstrate that a loop between activation and sensation exists even on the level of mere information intake. That is, the output of stimulus processing, i.e., perception, is fundamentally coupled to the state of the pupil, which adjusts the most basic visual information intake. Besides the possibility of other evolutionary accounts (see Douglas, 2018), Mathôt and Ivanov (2019) argue that a dilated pupil is beneficial for detection performance, which might be why pupil sizes change during strong emotional responses (e.g., during the fight-flight response). Here, we extend this view, by first replicating this finding and second demonstrating that peripheral detection performance is also increased with increasing changes in pupil size per se, i.e., not only the absolute size, but also the degree of change is decisive for high-detection performance. This might be why pupil sizes constantly co-vary with fluctuations in arousal and thus change size.

## Data Availability

The datasets generated and analyzed during the current study are available at the Open Science Framework repository: https://osf.io/ujbts/?view_only=b5dac82edd884c6bb861a74a6f8fdcec.
